# The Design of Near-Perfect Spectrum-Selective Mirror Based on Photonic Structures for Passive Cooling of Silicon Solar Cells

**DOI:** 10.3390/nano10122483

**Published:** 2020-12-10

**Authors:** Mengyu Gao, Ye Xia, Rong Li, Zhen Zhang, Yutian He, Chi Zhang, Laijun Chen, Lina Qi, Yang Si, Qinghong Zhang, Yuxiang Zheng

**Affiliations:** 1 New Energy (Photovoltaic) Industry Research Center, Qinghai University, Xining 810016, China; mygao16@fudan.edu.cn (M.G.); 18081291358@163.com (Y.X.); k3315657489@163.com (R.L.); zzok1005@163.com (Z.Z.); hugomax985@163.com (Y.H.); zhangchi4226@126.com (C.Z.); chenlaijun@tsinghua.edu.cn (L.C.); 2 Department of Optical Science and Engineering, Fudan University, Shanghai 200433, China; yxzheng@fudan.edu.cn; 3 State Key Laboratory for Modification of Chemical Fibers and Polymer Materials, Donghua University, Shanghai 201620, China; zhangqh@dhu.edu.cn

**Keywords:** selective-absorptive cooling, spectrally selective absorbing, radiative cooling, solar cells, nanostructures, optical multilayer film

## Abstract

When exposed to sunlight, crystalline silicon solar cells (CSSC) will not only generate electric energy but are also heated by solar radiation. Such a self-heating effect makes the working temperature of CSSC 20–40 °C higher than that of the ambient temperature, which degrades their efficiency and reliability. The elevated operating temperatures of CSSC are mainly derived from absorbing photons that cannot be converted to electrons. Therefore, it is important to prevent CSSC from absorbing useless solar light to have a better cooling effect. In this paper, photonic structures based spectrum-selective mirror is designed to cool the operating temperatures of CSSC passively. The mirror could make CSSC absorb about 93% of the sunlight in the wavelength range of 0.3 to 1.1 µm and only absorb about 4% of the sunlight in the wavelength range of 1.1 to 2.5 µm. Meanwhile, the design has good compatibility with the radiative cooling strategy. By applying selective-absorptive and radiative cooling strategies, the operating temperature of CSSC could be decreased about 23.2 K and 68.1 K under different meteorological conditions. Moreover, unlike the single radiative cooling strategy, the spectrum-selective mirror also has effective cooling effects in high wind speed meteorological conditions.

## 1. Introduction

By the end of 2019, the worldwide cumulative installed photovoltaic electricity generation capacity was over 635 GW [1]. Most photovoltaic panels rely on cells made from silicon crystals [2]. The conversion efficiencies of common crystalline silicon solar cells (CSSC) are about 20% [3]. This means only ~20% of incoming solar light could be converted to electricity, and ~80% of the sunlight may dissipate as heat in the photovoltaic panels, which causes a self-heating effect [4]. Thus, the operating temperatures of outdoor silicon solar panels may increase up to 50–55 °C [3]. Increased operating temperatures have adverse effects both on performance and reliability of CSSC: the relative efficiencies decrease by 0.25–0.45% with an increase in operating temperature of 1 °C [3,4,5,6,7,8,9], and the aging rate doubles when the operating temperature increases 10 °C [3,5,8]. Therefore, an attractive approach to increase conversion efficiencies and lifetimes of CSSC is to reduce the operating temperature through active and passive cooling methods.

A variety of impactful approaches have been utilized to cool the solar cells [8], including heat pipe cooling [10,11], water cooling [12,13,14,15], airflow cooling [16,17,18,19], jet impingement cooling [20] and phase change material cooling [21,22,23,24]. However, the aforementioned cooling methods need extra energy input and incur increasing costs, which makes them economically unfeasible. In recent years, achieving passive cooling effects by changing the spectral response of solar cells to electromagnetic waves has attracted much attention [25]. Research has shown that the self-heating effect of photovoltaic panels comes from incomplete thermal radiation and sub-bandgap absorption. Therefore, the fundamental principles of spectral selective cooling methods could be categorized as enhancing the thermal emission of solar cells and reducing the absorption of sub-bandgap photons (unable to generate electron–hole pairs). Herein, we named these two passive cooling methods as radiative cooling and selective-absorptive cooling, respectively.

The main principle of radiative cooling is to enhance the surface thermal emission so that the temperature of solar cells decreases. Some excellent studies of radiative cooling of solar cells have been reported [5,6,26,27] in recent years. The effect of radiative cooling is strongly dependent on the local topographic and meteorological conditions. The literature [28,29] showed radiative cooling is more effective in warm, arid conditions, especially in the presence of weak non-radiative cooling [5] (e.g., low wind speed). Nevertheless, the commercial solar cells already have a high thermal emissivity of about 0.8 because they are encapsulated with glass. Therefore, the study will be limited to improve the radiative cooling on the basis of glass-encapsulated solar cells [25]. Moreover, the radiative cooling effect varies with different topographic and meteorological conditions. For example, under the condition of strong non-radiation cooling (such as high wind speed), the radiation effect is not outstanding. Therefore, it is not an ideal passive cooling strategy for everywhere installed solar panels, and other effective passive cooling strategies should be figured out.

Because a part of elevated temperatures of photovoltaic panels is attributed to the parasitic sub-bandgap absorption, selective-absorptive cooling is proposed to prevent solar cells from absorbing sub-bandgap photons of solar light. Taking CSSC, for example, the photons with energy above the silicon bandgap in the wavelength range of 0.3 to 1.1 µm are expected to be absorbed by CSSC to generate electron–hole pairs, and the photons with energy below the silicon bandgap in the wavelength range of 1.1 to 2.5 µm are expected to be reflected by CSSC to decrease the waste-heat generation. However, actual photovoltaic panels have a large amount of absorption in the sub-bandgap wavelength range. Some outstanding studies of selective-absorptive cooling of solar cells have already been reported [3,4,25,30,31], which showed selective-absorption is an effective cooling method for different kinds of solar cells. However, because most selective-absorptive coolers are designed with multilayered optical films, it is difficult to make the cooler have low reflectivity in the wavelength range of 0.4–1.1 µm and high reflectivity in the wavelength range of 1.1–2.5 µm. For example, Li et al. [3] first proposed an effective selective-absorptive cooler that has very low reflectivity within 0.4–1.1 µm. Zhao et al. [7] designed a cooler that can make the reflectivity lower to about 4.5% in the wavelength range of 0.4–1.1 µm. Slauch et al. [30] have demonstrated the reflectivity of a cooler is lower than 0.1% in the wavelength range of 0.3–1.1 µm. However, the reflectivity in the wavelength range of 1.1–2.5 µm is not high enough in the aforementioned studies (~55% [3], ~56% [7], ~50% (at the range of 1.1–2.2 µm) [30]).

**Figure 1:**
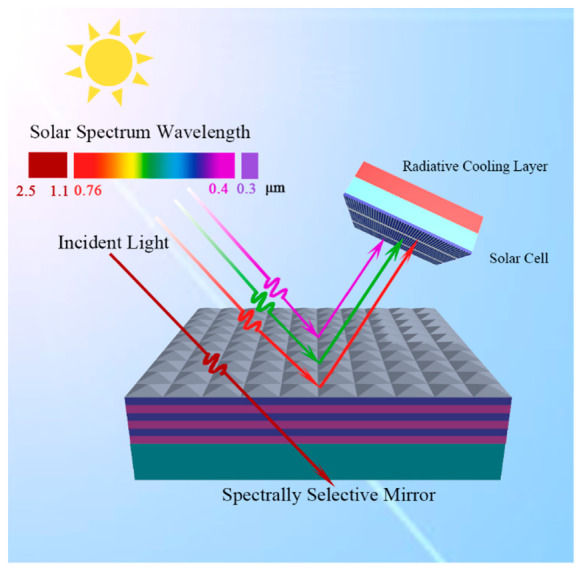
Schematic of the passive cooling strategy for crystalline silicon solar cells (CSSC).

Inspired by the design concept of the spectrum-selective mirror in literature [4], a near-perfect spectrum-selective mirror was designed in this report to cool CSSC, as shown in Figure 1. CSSC can be cooled by a spectrum-selective mirror through the following steps: the spectrum-selective mirror is irradiated by incident solar light; light with a wavelength range of 0.3–1.1 µm will be reflected onto CSSC and further generate electron–hole pairs, while light with a wavelength range of 1.1–2.5 µm will transmit spectrum-selective mirror and thus prevent CSSC from self-heating. Because a sky-facing terrestrial object can achieve the radiative cooling effect by adding radiative layers, a radiative cooling layer is placed on the sunlight-facing side of solar cells [3,5,6,7,8,9,30,31,32,33]. However, the radiative cooling layers could easily affect the light absorption of solar cells, and it is hard to optimize radiative cooling and selective-absorptive cooling effects at the same time. In this study, the backside of CSSC was placed toward the sky, which made the radiative cooling effect easily achieved. With the effect of radiative cooling and selective-absorptive cooling, CSSC was efficiently cooled both under strong and weak non-radiative cooling conditions.

## 2. Materials and Methods

### 2.1. Electromagnetic Simulation of Spectrum-Selective Mirror

**Figure 2:**
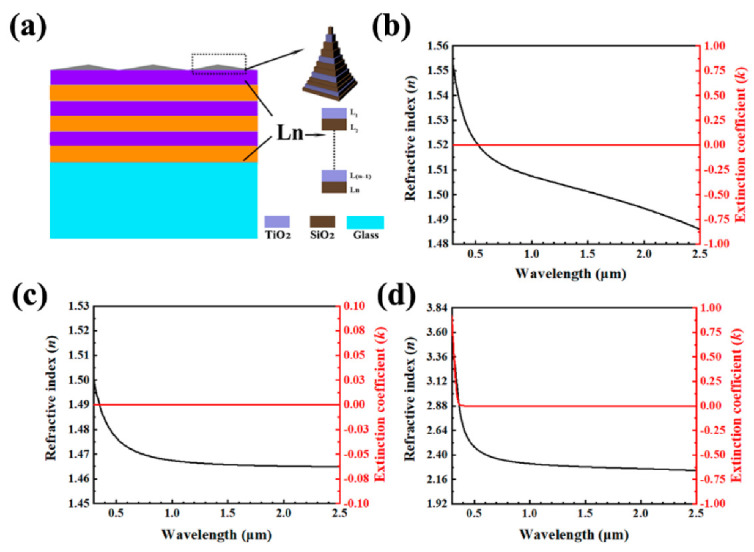
(**a**) Schematic of the photonic structure cooler made of a multilayer dielectric stack. The photonic cooler is designed on a glass substrate. The layers are made of SiO_2_/TiO_2_. The structure thickness is aperiodic for optimized performance. Optical constants of BK7 glass (**b**), SiO_2_ (**c**) and TiO_2_ (**d**).

FDTD solution software (Lumerical) was used to calculate the reflectance and electric field intensity ***E***. The spectrum-selective mirror, consisting of layers of SiO_2_/TiO_2_ with an aperiodic arrangement of thickness, was designed on a glass substrate. The mirror was made mainly of three parts, the top pyramidal structure, the middle optical multilayers and the bottom BK7 glass substrate. The cross-sectional view of the mirror is shown in the left part of Figure 2a. The top 12 layers formed the top pyramidal structures, one of which was briefly demonstrated by the diagram in the upper right corner of Figure 2a. The specific parameters and arrangement of TiO_2_ and SiO_2_ films are shown in Table S1. The middle part, which is represented by L1-Ln labeled in Figure 2a, consisted of 33 layers of aperiodically alternating SiO_2_/TiO_2_ with equal length and width but different film thicknessed. A perfectly matched layer (PML) was applied on the boundary of *z*-direction, and the periodical boundary conditions were applied in the *x*- and *y*- directions. Electric field intensity ***E*** was recorded by frequency-domain power monitor.

The optical constants of BK7 glass, SiO_2_ and TiO_2_ are shown in Figure 2b–d. The refractive index dispersion relation of materials are fitted in Equations (1)–(3), respectively.

(1) $$n^{2}=1+\frac{{1.03961290\lambda }^{2}}{\lambda^{2}-0.00600070}+\frac{{0.23179157\lambda }^{2}}{\lambda^{2}-0.02001794}+\frac{{1.01046984\lambda }^{2}}{\lambda^{2}-103.560691}$$

(2) $${\;n}^{2}=1+\frac{{0.71685787\lambda }^{2}}{\lambda^{2}-0.08735146^{2}}+\frac{{0.42793919\lambda }^{2}}{\lambda^{2}-0.08734869^{2}}+\frac{{6.44401027\lambda }^{2}}{\lambda^{2}-296.985583^{2}}$$

(3) $$n^{2}=1+\frac{{4.097660\lambda }^{2}}{\lambda^{2}-0.229041^{2}}$$

### 2.2. Thermal Simulation of CSSC

**Figure 3:**
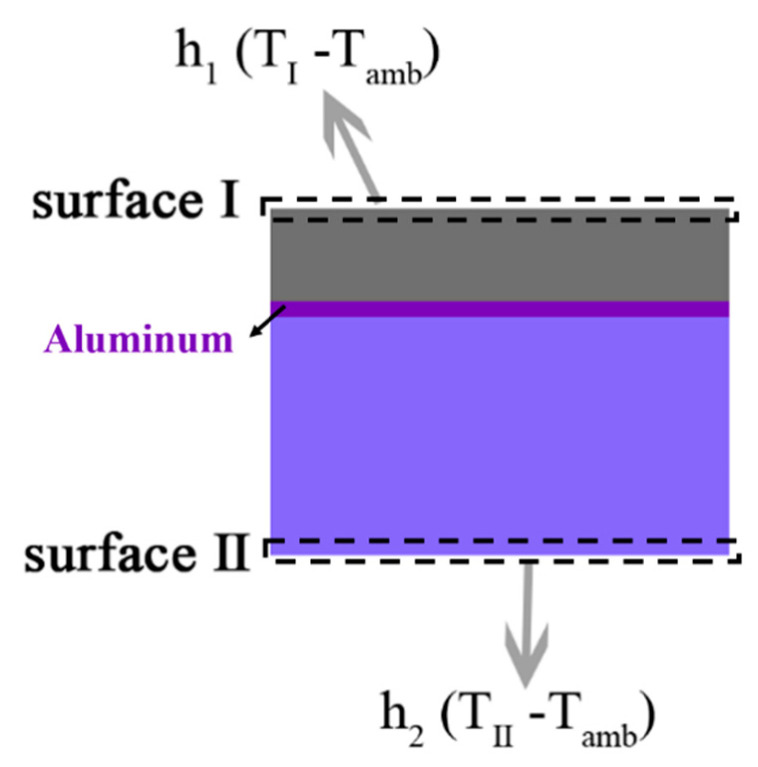
Schematic of thermal simulation for CSSC. $h_{1}$ and $h_{2}$ are the non-radiative heat transfer coefficients at the top and bottom surfaces, respectively.

The simulating model, which consists of 3 layers, was set up (as shown in Figure 3). The sky-facing surface I is integrated with the radiative cooling strategy, and the incident light reflected by the spectrally selective mirror is absorbed by surface II. The temperature distribution of CSSC is simulated by solving the steady-state heat diffusion equation:

(4) $$\frac{d}{dx}\left[k\left(x\right)\frac{dT\left(x\right)}{dx}\right]+\frac{d}{dy}\left[k\left(y\right)\frac{dT\left(y\right)}{dy}\right]+\frac{d}{dz}\left[k\left(z\right)\frac{dT\left(z\right)}{dz}\right]+\Phi =\;0$$

where *T* is temperature, *k* is thermal conductivity and Φ is the heat generation rate of CSSC. Here, we assume *k* is the isotropic and thermal conductivity of silicon, take aluminum and glass to be 130, 238 and 1.38 W/(m K), respectively. Heat generation rate Φ is corresponding to the difference between the absorbed solar power and extracted electrical power of CSSC (assume the power conversion efficiency of CSSC is 20%). The thermal boundary condition at the surface I is defined as:

(5) $$-k\left(z\right)\frac{dT\left(z\right)}{dz}{|}_{I}{=P}_{\mathrm{RC}}\left(T_{I}\right){\;+\;h}_{1}\left(T_{I}-T_{\mathrm{amb}}\right)$$

where $h_{1}$ is the non-radiative heat exchange coefficient, take $P_{\mathrm{RC}}\left(T_{I}\right)$ and $h_{1}\left(T_{I}-T_{\mathrm{amb}}\right)$ as radiative cooling and non-radiative heat dissipation (due to convection and conduction) effects. $P_{\mathrm{RC}}\left(T_{I}\right)$ is clearly defined in the literature [3,5]. The thermal boundary condition (6) characterizes the non-radiative heat loss of the surface II.

(6) $$k\left(z\right)\frac{dT\left(z\right)}{dz}{|}_{\mathrm{II}}{=\;h}_{2}\left(T_{\mathrm{II}}-T_{\mathrm{amb}}\right)$$

COMSOL Multiphysics software was used to calculate the operating temperature of CSSC in this study, and the operating temperature of CSSC was then defined as the spatially averaged temperature inside the solar cell region.

## 3. Results and Discussion

### 3.1. Simulated Reflectance of Spectrum-Selective Mirror

**Figure 4:**
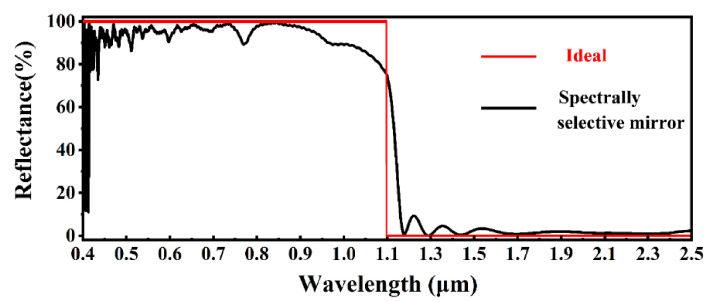
The reflectance of a spectrally selective mirror.

The reflectance of the designed spectrum-selective mirror is shown in Figure 4. The average reflectance in the wavelength range of 0.3 to 1.1 µm and 1.1 to 2.5 µm was about 93% and 4%, respectively. Because the design is based on the band-pass filter, the edge of the stopband is sharp and clear. The reflectance value drops evidently when the wavelength reaches to about 1.1 µm. The edge of the stopband matches well with the absorption cutoff wavelength of CSSC. Moreover, As Figure S1 shows, the stopband position of a spectrum-selective mirror could be adjusted to a shorter wavelength direction to adapt to other kinds of solar cells. By adjusting the length of the pyramid structure in Figure 2a, the average reflectance values of the spectrum-selective mirror with the length range of 0.05–4 µm are simulated in Figures S2 and S3. The result shows the spectra reflectance could be optimized by applying suitable pyramid structures on top of the mirror. The mirror also has a good performance when the length of the pyramid structure is extended. Thus, users could choose a relatively suitable way to get the passive cooling effect. Because only about 1% of solar irradiation is at wavelengths longer than 2500 nm, they are excluded from this study to simplify the calculation. The reflectance *R*(*λ*) with respect to solar photon flux density is shown in Figure S4. The result shows that most of the useful photons reflected by the mirror could get into CSSC and further generate electron–hole pairs, while most parts of useless photons are prevented from getting into CSSC to avoid the heating effect. The shape of the pyramidal structure also affects the performance of the mirror. The average reflectance values of the spectrum-selective mirror with different shapes of structures are shown in Table S2, which shows the average reflectance values of pyramidical, cylindrical, ring cylindrical, star and cross structures are almost the same in the wavelength range of 0.4–1.1 µm, while the average reflectance values in the wavelength range of 1.1–2.5 µm are evidently different. Because the refractive index is gradually varied in pyramidical structure, it has a better anti-reflection performance in the wavelength range of 1.1–2.5 µm.

**Figure 5:**
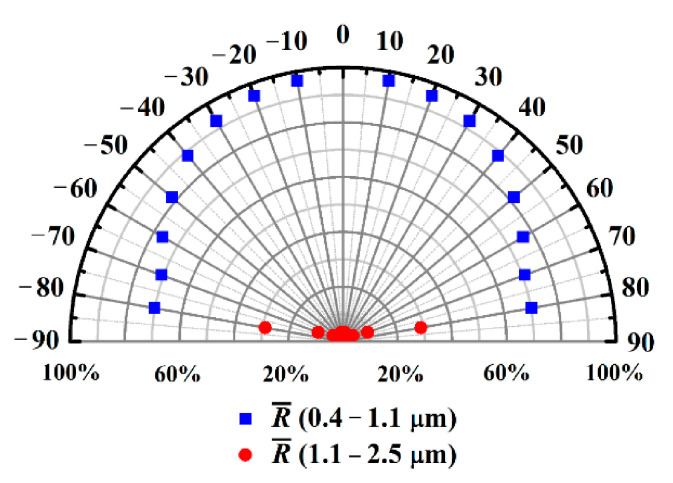
Simulated average reflectance of spectrum-selective mirror at different angles of incidence.

Outdoor installed solar panels generate electric powers at different incident angles of sunlight because of the rotation of the earth. The average reflectance values of the spectrum-selective mirror at different angles of incidence are shown in Figure 5. According to the literature [34], the average reflectance $\bar{R}$ is taken as the average values of $R_{TE}$ and $R_{TM}$ (as shown in Equation (7)). The simulated averaged-reflectance values $\bar{R}$ of the spectrum-selective mirror at different angles of incidence are shown in Figure 5.

(7) $$\bar{R}=\frac{R_{TE}+R_{TM}}{2}$$

where $R_{TE}$ and $R_{TM}$ are the averaged reflectance values for *TE* and *TM* mode, respectively. The spectrum-selective mirror has average reflectance values of 96.63%, 95.38%, 92.96%, 88.49%, 81.77%, 76.32%, 70.83% and 70.17% at incident angle of 10°, 20°, 30°, 40°, 50°, 60°, 70° and 80°, respectively, in the wavelength range of 0.4–1.1 µm. The results show the mirror could reflect the light effectively at incident angles range of 10 to 40°. Meanwhile, the mirror has average reflectance values of 3.34%, 2.71%, 2.23%, 1.87%, 2.68%, 4.16%, 9.62% and 28.98% at incident angles of 10°, 20°, 30°, 40°, 50°, 60°, 70° and 80°, respectively, in the wavelength range of 1.1–2.5 µm. The results show the most sunlight in the wavelength range of 1.1–2.5 µm could transmit the mirror until the incident angle reaches 70°. The simulated results of the oblique incidence of sunlight indicate that the installation system should be carefully designed when the spectrum-selective mirror is applied in reality.

**Figure 6:**
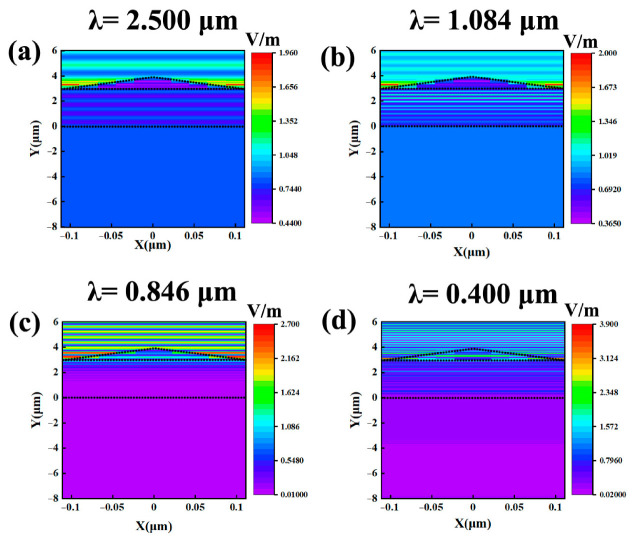
Distributions of electric field ***E*** with a normal incidence of light at incident wavelengths of 2.500 (**a**), 1.084 (**b**), 0.846 (**c**) and 0.400 (**d**) µm.

Distributions of electric field ***E*** with a normal incidence of light at different incident wavelengths are calculated and depicted in Figure 6. The electric field obviously exists in the glass substrate at an incident wavelength of 2.500 and 1.084 µm in Figure 6a,b. However, the electric field barely exists in the glass substrate at an incident wavelength of 0.846 and 0.400 µm in Figure 6c,d. The above results indicate that the mirror has an effective function to make the incident light at a wavelength of 2.5 and 1.084 µm transmit the mirror and reflect the incident light at a wavelength of 0.846 and 0.400 µm to the upper space.

### 3.2. Simulation of Cooling Effect with Different Solar Heating Powers

**Figure 7:**
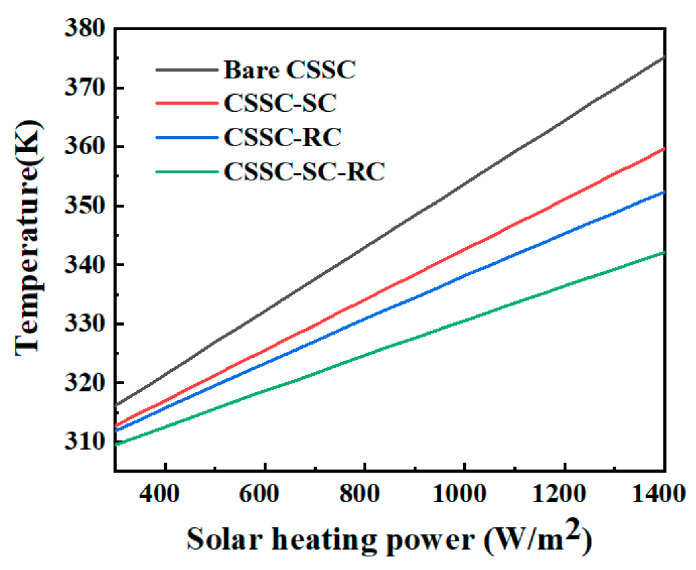
Calculated operating temperatures of bare CSSC, selective-absorptive cooler (CSSC-SC), CSSC with radiative cooler (CSSC-RC) and CSSC with both selective-absorptive cooler and radiative cooler (CSSC-SC-RC). The non-radiative heat exchange coefficients are $h_{1}\;$= 12 W/(m^2^ K), and $h_{2}\;$= 6 W/(m^2^ K). Both the ambient temperatures at the top and the bottom are 300 K.

The operating temperatures of bare CSSC, CSSC with selective-absorptive cooler (CSSC-SC), CSSC with radiative cooler (CSSC-RC) and CSSC with both selective-absorptive cooler and radiative cooler (CSSC-SC-RC) are calculated by setting up various incident solar heating powers. In particular, the non-radiative heat transfer coefficients of surface I and II are determined as $h_{1}$ = 12 W/(m^2^ K) and $h_{2}$ = 6 W/(m^2^ K), respectively (corresponding to the wind speeds of 3 m/s and 1 m/s [35]), and the ambient temperature is set up as 300 K to mimic a typical outdoor condition. In Figure 7, the operating temperature of bare CSSC increases considerably with the solar heating powers and could get to 353.7 K when the solar heating powers get to 1000 W/m^2^. However, CSSC applying selective-absorptive and radiative coolers could reduce the operating temperature by about 11.9 K and 15.6 K, respectively, with the solar heating power of 1000 W/m^2^. If the above two passive cooling strategies are applied at the same time, the cooling temperature could get to 23.2 K (As to the radiative cooling strategy, we have referred to our earlier work [9]).

### 3.3. Simulation of Cooling Effect with Different Non-Radiative Heat Exchange Coefficients

**Figure 8:**
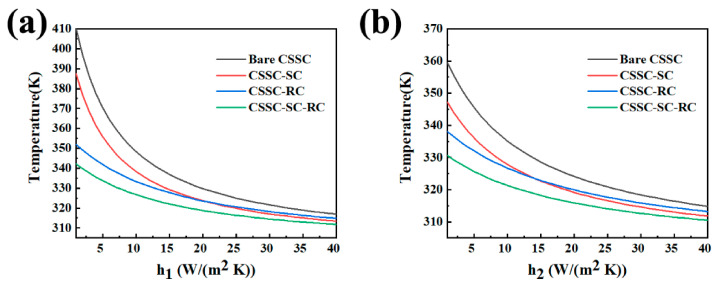
(**a**) Operating temperature of bare CSSC, CSSC-SC, CSSC-RC and CSSC-SC-RC with different $h_{1}$, and fixed $h_{2}\;$= 6 W/(m^2^ K). (**b**) Operating temperature of bare CSSC, CSSC-SC, CSSC-RC and CSSC-SC-RC with different $h_{2}$, and fixed $h_{1}\;$= 12 W/(m^2^ K). Both the ambient temperatures at the top and the bottom are 300 K.

The operating temperatures of bare CSSC, CSSC-SC, CSSC-RC and CSSC-SC-RC as a function of non-radiative heat transfer coefficient at the surface I or II are calculated in Figure 8a,b, respectively. It is possible to visualize that selective-absorptive cooling and radiative cooling strategies have stable cooling effects when $h_{1}$ and $h_{2}$ values vary in the range of 1–40 W/(m^2^ K). Compared to radiative cooling, selective-absorptive cooling is more effective when $h_{1}$ or $h_{2}$ value is higher than 21 and 12 W/(m^2^ K), respectively. Furthermore, the combined application of the two cooling strategies could achieve a better cooling effect. The temperature difference between CSSC-SC-RC and bare CSSC is 68.1 K when surface I has a weak non-radiative heat transfer coefficient ($h_{1}$ = 1 W/(m^2^ K), the wind speed is 0 m/s), while the difference is 5.1 K, when surface I has a strong non-radiative heat transfer coefficient ($h_{1}$ = 40 W/(m^2^ K), the wind speed is 12.4 m/s). Accordingly, the cooling is effective no matter when $h_{1}\;$= 1 W/(m^2^ K) (the wind speed is 0 m/s) or $h_{1}\;$= 40 W/(m^2^ K) (the wind speed is 12.4 m/s). The simulation results obtained above have greatly improved the working temperature of CSSC. According to the literature [36,37], the operating temperature of CSSC is dependent on the light reflection of the surface, which could be estimated from the following equation:

(8) $$\left(\tau \alpha \right)G=\eta G+U_{L}\left(T_{c}-T_{a}\right)$$

where *τ* is the cell cover transmittance, which could be represented as 1-*R* when the cover does not absorb the sunlight, and *R* is the reflectance of the cell cover. The absorptance and efficiency of the cell are represented as *α* and *η*, respectively. *G* is the solar radiation, *T*_c_ is the cell temperature, and *T*_a_ is the ambient temperature. The loss coefficient *U*_L_ includes losses by convection and radiation from top and bottom and by conduction. Thus, reducing the surface reflectance of CSSC could effectively reduce its operating temperature. In the CSSC industry, it is crucial that the absolute efficiency is only improved by one percent. Scientists had spent about 10 years to make the record efficiency of CSSC module increased by 1.1 percent (22.7% in the year 2006 [38] and 23.8% in the year 2016 [39]). Because the operating temperatures of CSSC influence panels’ efficiencies (the rising of one centigrade makes the panel’s relative efficiency decline by ~0.45% [3,4,26,40]). The cooling strategy is a new path to enhance the working efficiency of CSSC. Herein, the temperature of CSSC-SC-RC is 68.1 K lower than that of bare CSSC under $h_{1}\;$= 1 W/(m^2^ K) and $h_{2}\;$= 6 W/(m^2^ K), which could provide an absolute efficiency improvement of about 6.7% for CSSC. Even under the meteorological condition of high wind speed ($h_{1}\;$= 40 W/(m^2^ K) and $h_{2}\;$= 6 W/(m^2^ K), the temperature reduction of 5.1 K could also provide an absolute efficiency improvement of about 0.5% for CSSC.

Selective-absorptive cooler and radiative cooler has different cooling temperatures. Simulation results show that the relative cooling effect of a radiative cooler is better than that of the selective-absorptive cooler under the same meteorological conditions. However, the radiative cooling effect is limited since solar cells were encapsulated with glass, which already has a radiative cooling effect. Consequently, selective-absorptive cooling has become an effective strategy. Moreover, as is well-known, radiative cooling has poor performance under the high wind speed outdoor circumstance; other passive cooling strategies are welcomed in such topographic and meteorological conditions. The selective-absorptive cooling strategy is independent of topographic and meteorological conditions and thus is efficient when radiative cooling is invalid. Moreover, selective-absorptive cooling and radiative cooling could be combined together in this study without any interaction. It should be noted that the performance of solar cells is affected by the oblique illumination of light. Although the spectrum-selective mirror could improve the efficiency of solar cells by cooling, the spectrum-selective mirror should be carefully installed according to the incidence sunlight angle.

## 4. Conclusions

In order to reduce the operating temperature of CSSC without any additional energy input, a spectrally selective reflector is designed. The spectrum-selective mirror with nearly perfect spectral absorption property has an average reflectance of about 93% in the wavelength range of 0.3 to 1.1 µm and about 4% in the wavelength range of 1.1 to 2.5 µm. In this way, the design prevents CSSC from absorbing useless photons that cannot generate electron–hole pairs. Moreover, it applies a radiative cooling strategy without altering the light absorption of CSSC to further enhance the thermal emission of solar cells, making it has potent cooling effects under both low and high wind speed meteorological conditions. Moreover, the operating temperature of CSSC could be reduced by 23.2 K under solar heat power of 1000 W/m^2^. Therefore, by combining two highly potential cooling technologies, selective-absorptive cooling and radiative cooling, together, the design becomes a passive cooling method with superior performance.

## Supplementary Materials

The following are available online at https://www.mdpi.com/2079-4991/10/12/2483/s1, Figure S1: The simulated reflectance of a spectrum-selective mirror with a cutoff wavelength of 0.8 µm. Figure S2: Averaged reflectance of the spectrum-selective mirror with the periods of pyramidal structures vary from 0.05–4 µm. Figure S3: Averaged reflectance of the spectrum-selective mirror with the periods of pyramidal structures vary from 0.05 –0.5 µm. Figure S4: Normalized reflectance of a spectrum-selective mirror with respect to solar photon flux density. Table S1: Layer thickness parameters for various photonic cooler designs. Table S2: The average reflectance values of spectrum-selective mirror with different shapes of structures.
