# Supplementary material for: The Design of Near-Perfect Spectrum-Selective Mirror Based on Photonic Structures for Passive Cooling of Silicon Solar Cells
Source: Nanomaterials (Basel). 2020 Dec 10;10(12):2483. doi: 10.3390/nano10122483 (PMC7763757; doi:10.3390/nano10122483)
Supplement: Supplementary file 1 [file nanomaterials-10-02483-s001.pdf]

Supplementary Materials: The Design of Near-Perfect Spectrum-Selective Mirror Based on Photonic Structures for Passive Cooling of Silicon Solar Cells

Mengyu Gao [1,2,†], Ye Xia [1,†], Rong Li [1], Zhen Zhang [1], Yutian He [1], Chi Zhang [1], Laijun Chen [1], Lina Qi [1,*], Yang Si [1,*], Qinghong Zhang [3] and Yuxiang Zheng [2]

1. The Simulated Reflectance of Spectrum-Selective Mirror with Cutoff Wavelength of 0.8 μm

The drop position of the reflectance spectrum could be adjusted to a shorter wavelength direction as shown in Figure S1.

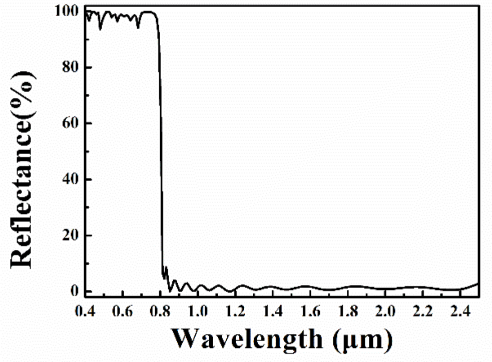

Figure S1. The simulated reflectance of the spectrum-selective mirror with cutoff wavelength of 0.8 μm.

2. Simulated Reflectance of the Spectrum-Selective Mirror with Different Length of Pyramidal Structures

By adjusting the length of the pyramid structure, the averaged reflectance values of the spectrum-selective mirror with length range of 0.05–4 μm is simulated in Figure S2 and Figure S3. The results show that the mirror still has a good performance when the length of the pyramid structure is extended.

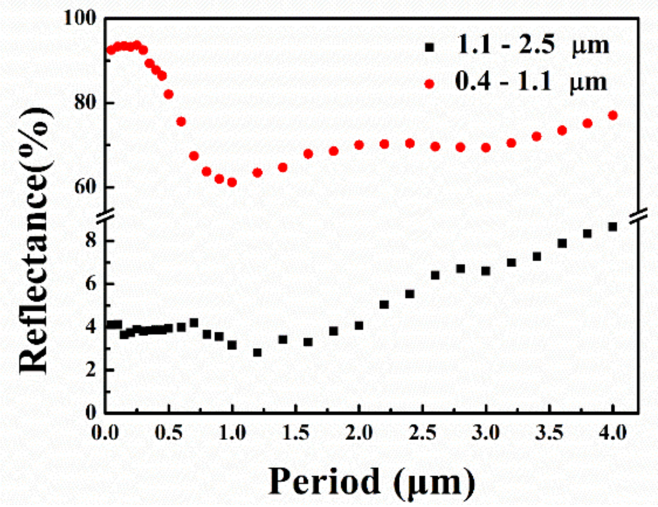

Figure S2. Averaged reflectance of the spectrum-selective mirror with the periods of pyramidal structures vary from 0.05–4 μm.

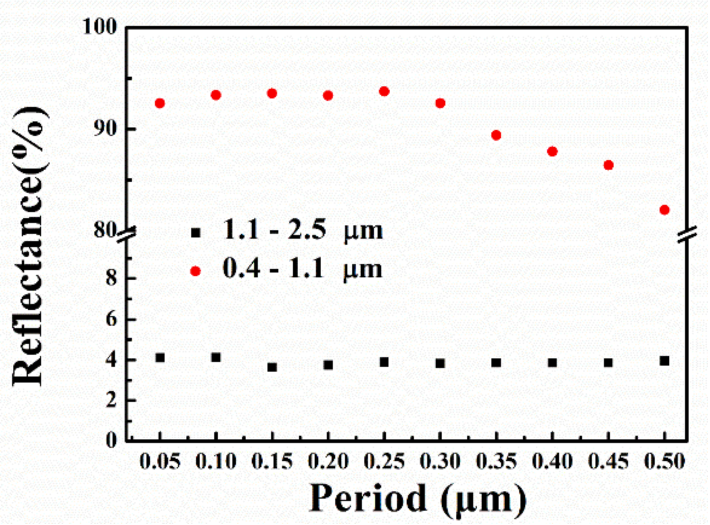

Figure S3. Averaged reflectance of the spectrum-selective mirror with the periods of pyramidal structures vary from 0.05–0.5 μm.

3. Chromatic Dispersion Characteristics of Reflectivity with Respect to Solar Photon Flux Density

The reflectance $R(\lambda)$ with respect to solar photon flux density is shown in Figure S4. The results show that most solar energy which has wavelength range of 0.4–1.1 μm could be reflected to CSSC, and most solar energy which has wavelength range of 1.1–2.5 μm would transmit the spectrum-selective mirror to prevent heating CSSC.

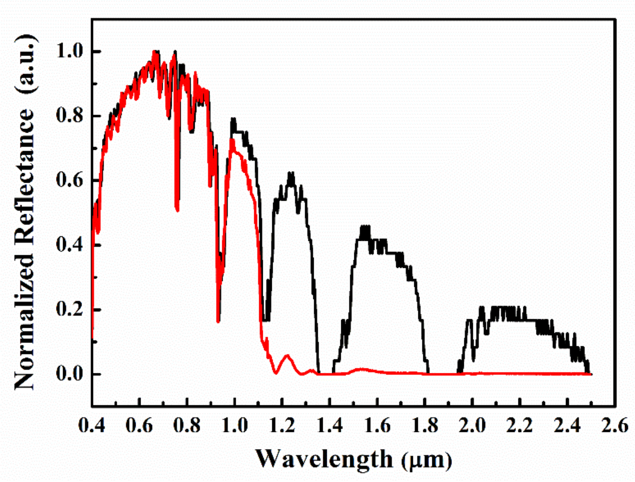

Figure S4. Normalized reflectance of the spectrum-selective mirror with respect to solar photon flux density.

4. Specific parameters of the spectrum-selective mirror

Table S1. Layer thickness parameters for various photonic cooler designs.

Pyramidal structure (constructed by top 12 layers)				
**Layer Number**	**Materials**	**Width (nm)**	**Length (nm)**	**Height (nm)**
1	SiO_2	17	17	65
2	TiO_2	33	33	104
3	SiO_2	50	50	66

4	SiO_2	67	67	66
5	TiO_2	83	83	104
6	SiO_2	100	100	66
7	SiO_2	117	117	66
8	TiO_2	133	133	104
9	SiO_2	150	150	96
10	SiO_2	167	167	91
11	TiO_2	183	183	112
12	SiO_2	200	200	89

Layer Number	Materials	Thickness (nm)	Layer Number	Materials	Thickness (nm)
13	SiO_2	85	30	TiO_2	62
14	TiO_2	103	31	SiO_2	108
15	SiO_2	158	32	TiO_2	63
16	TiO_2	98	33	SiO_2	92
17	SiO_2	158	34	TiO_2	53
18	TiO_2	104	35	SiO_2	95
19	SiO_2	151	36	TiO_2	51
20	TiO_2	91	37	SiO_2	80
21	SiO_2	138	38	TiO_2	46
22	TiO_2	73	39	SiO_2	88
23	SiO_2	116	40	TiO_2	39
24	TiO_2	72	41	SiO_2	50
25	SiO_2	138	42	TiO_2	46
26	TiO_2	82	43	SiO_2	67
27	SiO_2	131	44	TiO_2	43
28	TiO_2	67	45	SiO_2	19
29	SiO_2	102			

5. Average Reflectance Values of the Spectrum-Selective Mirror with Different Shapes of Structures

Table S2. The average reflectance values of spectrum-selective mirror with different shapes of structures.

Shape	Schematic	Average Reflectance (0.4–1.1μm)	Average Reflectance (1.1–2.5μm)
Pyramidical structure	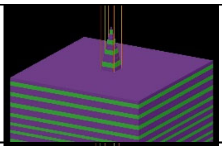	93%	4%
Cylinder	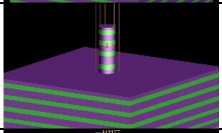	92%	10%
Ring cylinder	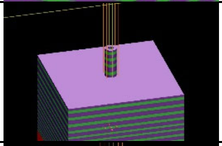	91%	8%
Star	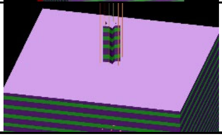	91%	13%

Cross	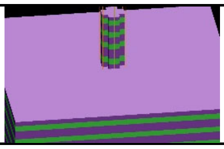	92%	11%
